# Rapid Development of Th2 Activity During T Cell Priming

**DOI:** 10.1080/10446670310001598537

**Published:** 2003-03

**Authors:** Adam F. Cunningham, Kai-Michael Toellner

**Affiliations:** University of Birmingham Medical Research Council Centre for Immune Regulation Birmingham B15 2TT UK

## Abstract

The paradigm of T helper-1 (Th-1) and Th-2 cells developing from non-committed naïve precursors is firmly established. Th1 cells are characterized by IFN production and, in mice, the selective switching to IgG2a. Conversely IL-4 production and selective switching to IgG1 and IgE characterize Th2 cells. Analysis of Th2 induction *in vitro* indicates that this polarization develops gradually in T cells activated by anti-CD3 in the presence of IL-4; conversely anti-CD3 and IFN induce Th1 cells. In this report, we explore evidence that indicates that the T helper cell polarization *in vivo* cannot solely be explained by the cytokine environment. This is provided by studying the early acquisition of Th1 and Th2 activities during responses to a mixture of Th1 and Th2-inducing antigens. It is shown that these divergent forms of T cell help can rapidly develop in cells within a single lymph node. It is argued that early polarization to show Th-1 or Th-2 behavior can be induced by signals delivered during cognate interaction between virgin T cells and dendritic cells, in the absence of type 1 or type 2 cytokines. This contrasts with the critical role of the cytokines in reinforcing the Th-phenotype and selectively expanding T helper clones.


*Clinical & Developmental Immunology*, March 2003, Vol. 10 (1), pp. 1–6

Taylor & Francis healthsciences

### Rapid Development of Th2 Activity During T Cell Priming

ADAM F. CUNNINGHAM and KAI-MICHAEL TOELLNER*

*University of Birmingham, Medical Research Council Centre for Immune Regulation, Birmingham B15 2TT, UK*

The paradigm of T helper-1 (Th-1) and Th-2 cells developing from non-committed naïve precursors is firmly established. Th1 cells are characterized by IFNγ production and, in mice, the selective switching to IgG2a. Conversely IL-4 production and selective switching to IgG1 and IgE characterize Th2 cells. Analysis of Th2 induction *in vitro* indicates that this polarization develops gradually in T cells activated by anti-CD3 in the presence of IL-4; conversely anti-CD3 and IFNγ induce Th1 cells. In this report, we explore evidence that indicates that the T helper cell polarization *in vivo* cannot solely be explained by the cytokine environment. This is provided by studying the early acquisition of Th1 and Th2 activities during responses to a mixture of Th1 and Th2-inducing antigens. It is shown that these divergent forms of T cell help can rapidly develop in cells within a single lymph node. It is argued that early polarization to show Th-1 or Th-2 behavior can be induced by signals delivered during cognate interaction between virgin T cells and dendritic cells, in the absence of type 1 or type 2 cytokines. This contrasts with the critical role of the cytokines in reinforcing the Th-phenotype and selectively expanding T helper clones.

*Keywords*: B lymphocytes; IL-4; T cell priming; Th2 differentiation

### INTRODUCTION

The host's response to danger occurs at two levels. The first level is innate where pathogen associated molecular patterns are recognized by phagocytes through activation of a range of receptors which are exemplified by the toll-like receptors (TLRs) (Janeway and Medzhitov, 1998). The TLRs recognize molecules expressed by pathogens such as lipopolysaccharide (TLR4), peptidoglycan (TLR2), flagellin (TLR5) and unmethylated DNA (TLR9) (Medzhitov, 2001). Once activated by innate immune signals dendritic cells take up and process antigen. This is associated with their differentiation to become antigen presenting cells, which migrate to secondary lymphoid tissues where they present processed peptides to recirculating T cells. This process of differentiation to antigen-presenting cells results in the stabilization of peptide:MHCII complexes and the upregulation of co-stimulatory molecules such as CD40, Ox-40 ligand, CD80, and CD86. In the T zone of the secondary lymphoid tissue the dendritic cell can interact with T cells that specifically recognize the peptide:MHCII complex on the dendritic cell (Guermonprez *et al.*, 2002). In responses to Th-2 inducing antigens the activation of T cells is marked by the induction to proliferate and upregulation of IL-4 production. In lymph nodes emerging Th-2 cells rapidly acquire the ability to attract B cells that have taken up antigen and induce selective switch recombination to IgG1. The switching process is heralded by the early production of sterile switch transcripts (Toellner *et al.*, 1998). The B cells activated in this way, either migrate to follicles where they form germinal centers, or to medullary cords where they proliferate as plasmablasts before differentiating into plasma cells (Luther *et al.*, 1997). Conversely B cells activated by emerging T helper-1 (Th-1) cells selectively switch to IgG2a (Coffman *et al.*, 1993). In this report, we summarize findings generated in our investigations of the response of mice after immunization with the Th-2 antigen—haptenated-chicken gamma globulin (NP-CGG) and the Th-1 antigen—heat-killed *B. pertussis* (pertussis) (Toellner *et al.*, 1998). There is a clear adjuvant effect on the response to NP-CGG when this is given with pertussis, but the switching in the NP and CGG-specific response remains almost exclusively to IgG1. Thus, when this Th-1 antigen is given with the Th-2 antigens the T helper polarization of the response to each antigen is remarkably similar to that developing in responses to the antigens given alone.

### MATERIALS AND METHODS

### Mice and Immunizations

Six to eight week old BALB/c mice were purchased from Harlan (Bicester, UK) and kept in isolators under sterile conditions.

*Corresponding author. Tel.: +44-121-414-6970. Fax: +44-121-414-3599. E-mail: K.M.Toellner@bham.ac.uk

ISSN 1740-2522 print/ISSN 1740-2530 online © 2003 Taylor & Francis Ltd
DOI: 10.1080/10446670310001598537

Alum-precipitated NP-CGG was prepared as previously described (Nossal and Karvelas, 1990; Luther *et al.*, 1997). Adult mice (8–12 weeks) were injected into both rear footpads with 20 μg alum-precipitated NP-CGG plus $5 \times 10^8$ heat killed *B. pertussis* (Evans Medical, Liverpool, UK). A 2 mg of BrdU was administered i.p. 2 h before sacrifice as previously described (Gulbranson-Judge and MacLennan, 1996).

### Tissue Preparation

After termination, blood was obtained for the preparation of sera and draining popliteal lymph nodes removed. Frozen lymph nodes were sectioned as described elsewhere (Toellner *et al.*, 1998). Five μm thick sections were cut for immunohistology, and mounted onto four spot glass slides. For mRNA extraction 25 μm thickness sections were cut, placed in polypropylene tubes and stored at −70°C. Glass mounted sections were air-dried for an hour, fixed in acetone (20 min, 4°C), air dried for 10 min and sealed in polythene bags at −20°C until use.

### Immunohistological Reagents, Staining and Analysis

Immunohistological reagents and staining was as described earlier (Luther *et al.*, 1997; Toellner *et al.*, 1998).

Cells were triple stained for CD3, IgD and BrdU. Rat anti-CD3 (Serotec, Oxford, UK) was labeled with biotinylated rabbit anti-rat antibodies (Dako, High Wycombe, UK) and sheep anti-mouse IgD (The Binding Site, Birmingham, UK) was labeled with peroxidase-labeled donkey anti-sheep (The Binding Site). After washing, StreptABComplex-alkaline phosphatase (Dako) was added to sections with biotin-conjugated antibodies. Horseradish peroxidase was detected using diaminobenzidine tetrahydrochloride solution (Luther *et al.*, 1997). Alkaline phosphatase activity was detected using naphthol AS-MX phosphate and Fast Blue salt with levamisole (Luther *et al.*, 1997). BrdU positive cells were detected as previously described (Gulbranson-Judge and MacLennan, 1996). After color development for CD3 and IgD staining cells were washed and treated for 20 min with 1M HCl at 60°C. BrdU containing cells were identified by labeling with a mouse anti-BrdU (Dako) primary antibody and secondarily labeled with a biotinylated goat anti-mouse antibody and StreptABComplex-alkaline phosphatase (Dako) added. Color was developed as described above with the exceptions that TBS pH8.2 and Fast Red TR salt (Sigma) were used. The surface area of lymph nodes and was determined using the point counting technique of Weible (Weible, 1963).

### Reverse Transcription of mRNA and its Relative Quantification by PCR

Polypropylene tubes containing lymph node sections were removed from the freezer directly onto ice. Cells were lysed directly and RNA extracted using RNAzol B (Biogenesis, Poole, UK) according to protocol. The RNA pellet was resuspended in 10 mM Tris, 0.1 M EDTA, (TE) buffer (pH 8.0) containing 1 μg of oligo-$dT_{12-18}$ (Amersham Pharmacia Biotech, High Chalfont, UK) and denatured at 70°C for 10 min. RNA was reverse transcribed using MMLV reverse transcriptase (Invitrogen, Paisley, UK) in the presence of 0.01 M dithiothreitol, 0.5 mM deoxynucleotide triphosphates, 1× first strand buffer and ≈20 U of RNase inhibitor (Amersham Pharmacia Biotech) at 42°C for 60 min. Reverse transcriptase was inactivated by heating to 90°C for 10 min. Finally, the cDNA was diluted to 100 μl with TE buffer.

Levels of IL-4, IFN-γ, γ1 switch transcript and γ2a switch transcript were determined using a semi-quantitative non-fluorescence technique (Toellner *et al.*, 1998).

In the non-fluorescence method mouse β-actin-specific primer sequences were obtained from Stratagene (Cambridge, UK). IL-4 and IFN-γ-specific primers were as described by Svetic *et al.* (1991). Other intron-spanning primers were designed using OLIGO version 5.0 (National Bioscienses, Plymouth, MA). γ1 switch

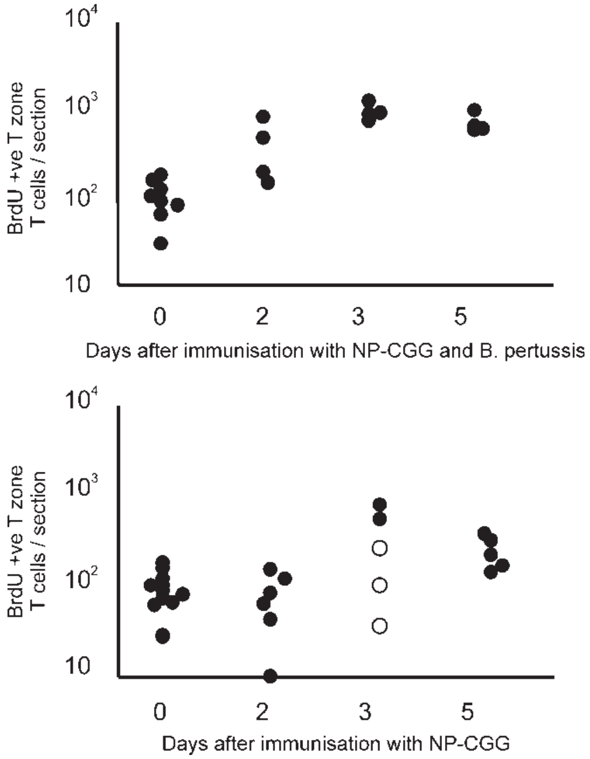

FIGURE 1 The induction of T cells to proliferate after immunization with NP-CGG and *B. pertussis* is an early event. Popliteal lymph nodes were recovered at pre-determined times from mice non-immunized mice and mice immunized with NP-CGG and *B. pertussis* (top panel) and NP-CGG alone (bottom panel). Number of T cells in the T zone are shown that had taken up BrdU during a 2 h pulse before the lymph node was taken. Each point equals one mouse. Three of the five mice immunized with alum precipitated NP-CGG alone 3 d earlier showed T zone T cell proliferation levels that were at or near background levels. The values for these three mice in this and subsequent figures are shown in open circles.

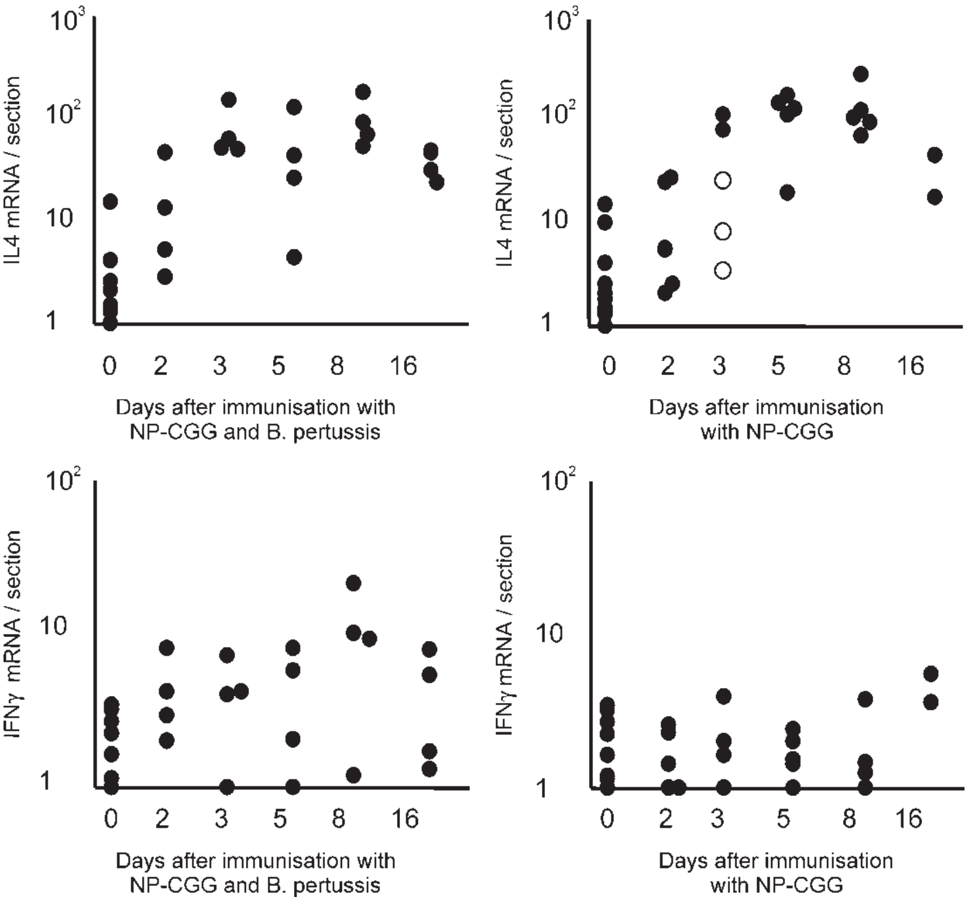

FIGURE 2 IL-4 and IFN-γ upregulation in response to immunisation with NP-CGG, with or without *B. pertussis*. Differential induction of IL-4 and IFN-γ mRNA in groups of mice immunized with alum-precipitated NP-CGG with (left hand panels) or without (right hand panels) *B. pertussis*. Amounts of switch transcript per lymph node section were determined by semiquantitative RT-PCR. The open circles indicate the mice which did not show T cell proliferation in response to NP-CGG alone.

transcript specific primers were (CCTCCTAGACAAG-CACAGGCATGTAGA) and (ACCATGGAGTTAGTT-TGGGCAGCAG) specific for the first exon of γ1 switch transcript and the first exon of γ1 constant region. Primers specific for γ2a switch transcript were (GTGC-CTACCTGCAGCCTGGGAT) located in exon 1 upstream of the first splice site of the γ2a switch transcript (Collins and Dunnick, 1993) and (CACTGA-CCACCCGGAGAGTACTGTTG) located in the γ2a constant region. Primers were synthesized by Life Technologies (Paisley, UK).

To quantitate cDNAs by Southern blot analysis about 10 fewer cycles than would be required to detect the PCR product using ethidium bromide gels were done, this was determined in preliminary experiments. For each cDNA sample 3 amplifications at different cycle numbers were made to improve precision and check that amplification was logarithmic in the range of cycle numbers used. PCR was performed with 2 μl cDNA template in 20 μl volume. For amplification of β-actin and γ1 switch transcript 0.1 μl Taq-Polymerase (Promega, Madison, WI) per reaction was mixed with TaqStart antibody (Clontech, Palo Alto, CA) 1:1 5 min before use. All other cDNAs were amplified using 0.1 μl AmpliTaq Gold (Perkin Elmer, Langen, Germany) per reaction. Buffers were used as supplied with the enzymes, plus 1 μM of each primer, 200 μM of each dNTP and 2.0 mM $MgCl_2$ for γ1 switch transcript, 2.75 mM $MgCl_2$ for γ2a switch transcript or 1.5 mM $MgCl_2$ for the other cDNAs. The reaction mix was overlaid with one drop of mineral oil (Sigma, Poole, England). The 3 PCRs were performed in 0.2 ml 96-well polypropylene plates in the 3 blocks of a Touch Down thermal cycler (Hybaid, Middlesex, UK). Cycling was done with an initial denaturation step of 2 min for Taq-Polymerase or 9 min for AmpliTaq Gold, followed by 30 s at 94°C, 30 s at annealing temperature (60°C for β-actin, 65°C for switch transcripts, and 50°C for cytokine mRNA) and 2 min plus 2 s for every cycle at 72°C (3 min for γ2a switch transcript). Cycle numbers were 16 ± 2 for β-actin, 24 ± 2 for both switch transcript, 28 ± 2 for IL-4 mRNA and 31 ± 2 for IFN-γ mRNA. The PCR product was separated on a 1.5% agarose gel and transferred onto a pre-wetted Hybond-N + membrane (Amersham International, Little Chalfont, UK) by capillary transfer under alkaline conditions. The membrane was hybridized with a $^{32}$P-labeled purified PCR product from an earlier PCR

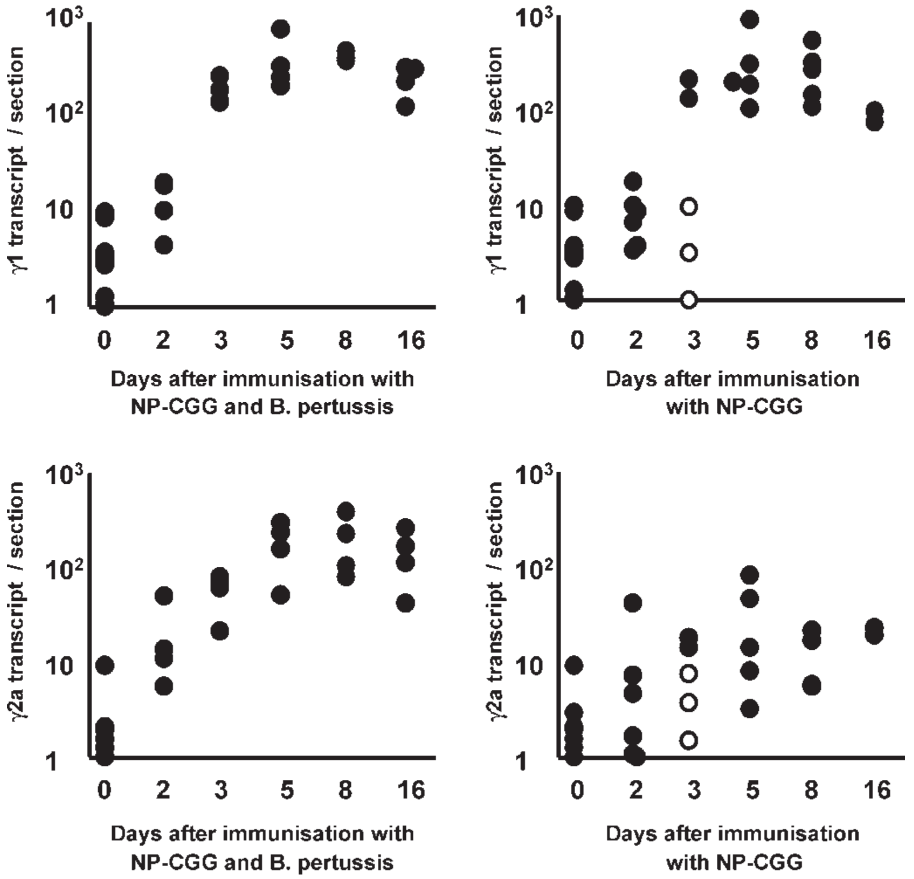

FIGURE 3 Differential induction of γ1 and γ2a switch transcript in groups of mice immunized with alum-precipitated NP-CGG with or without *B. pertussis*. Symbols show the amount of switch transcript per lymph node section of individual mice determined by semiquantitative RT-PCR. The open circles indicate the mice that did not show T cell proliferation in response to NP-CGG alone.

as a probe as described earlier (Toellner *et al.*, 1996) and imaged using a phosphorimager (Molecular Dynamics, Kent, UK).

Using the ImageQuant software (Molecular Dynamics) a grid was laid over the PCR bands, with individual fields covering the central 50% of a band. The signal in each field was calculated and these figures transferred to a spreadsheet software to sort the randomized figures to the correct order. The average of the 3 PCRs with different cycle number for each gene was taken and divided by the average of the 3 corresponding β-actin PCRs. These values are equivalent to the relative amount of mRNA for a gene per cell. This was multiplied with the section area, determined by microscopy on adjacent sections using the point counting technique (Weible, 1963), to give the mRNA amount per section.

PCR specific for β-actin, γ1 switch transcript, IL-4, and IFN-γ mRNA resulted in single bands and PCR for γ2a switch transcript in 3 bands of the expected size (Collins and Dunnick, 1993). Identity of the PCR products was confirmed by DNA sequencing (Alta Biosciences, Birmingham, UK), using the PCR primers as sequencing primers. The data were controlled for any signs of saturation of the PCR reaction between different cycle numbers. This was done by plotting PCR data from the 3 PCRs for a particular gene and checking for variations in the efficiency of the PCR amplification over different cycle numbers in samples containing a high amount of target cDNA with samples having low content of target cDNA. The amplification was shown to be logarithmic within the range of cycle numbers used.

### RESULTS

### Pertussis has an Adjuvant Effect on the Response to Alum-precipitated NP-CGG

Groups of BALB/c mice were immunized in a hind foot pad with ether NP-CGG or NP-CGG plus pertussis. The response in the draining popliteal lymph node was then assessed at intervals following immunization. The end points used were: (a) the onset of T cell proliferation in the T zone (Fig. 1), (b) the up-regulation of message for the type-2 cytokine IL4 and the type-1 cytokine γIFN assessed by RT-PCR on RNA extracted from the lymph node tissue (Fig. 2), (c) the levels of γ1 and γ2a switch transcripts induced (Fig. 3), (d) the total number of IgG1 and IgG2a-plasma cells produced (Fig. 4) and (e) the numbers of NP-specific plasma cells expressing this IgG1 or IgG2a (Fig. 5). The time of onset of T cell proliferation in the T zone in mice immunized with alum NP-CGG with pertussis is faster than in the response to NP-CGG alone.

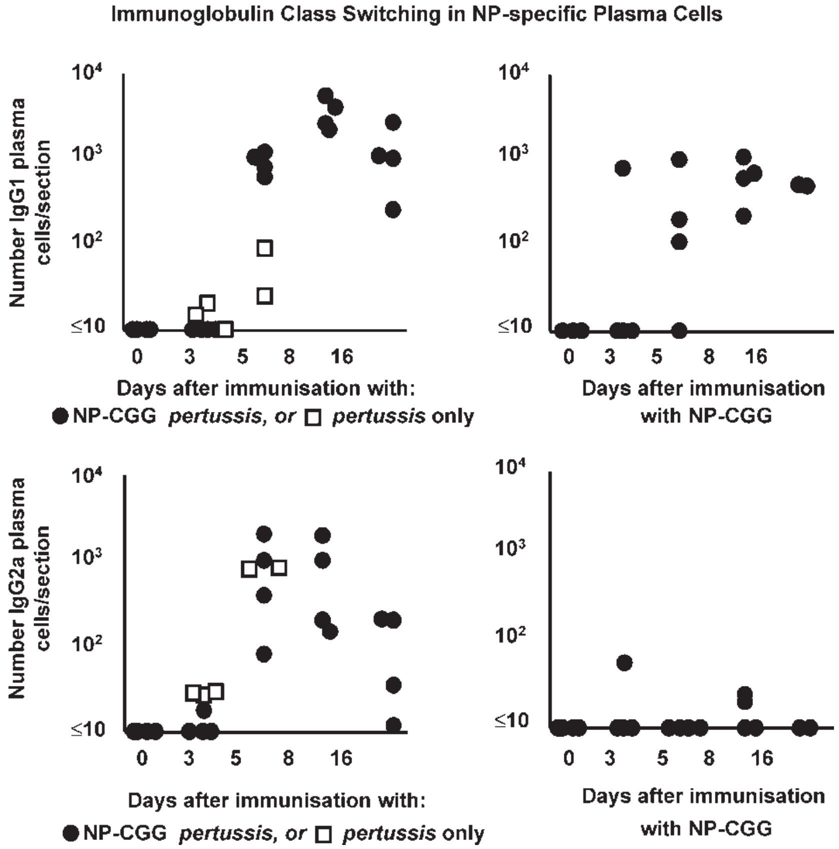

FIGURE 4 Numbers of NP-specific plasmablasts and plasma cells switched to IgG1 (top) or IgG2a (bottom) in the responses to NP-CGG with *pertussis*, *pertussis* only, or NP-CGG in alum without *pertussis*. Each point represents a single node.

There is a trend towards earlier upregulation of transcription of IL-4 message in the group immunized with NP-CGG plus pertussis, but this difference is not significant. The most convincing adjuvant effect of pertussis on the response to NP-CGG is seen with the induction of γ1 switch transcripts and the number of IgG1 plasma cells. Pertussis by itself induces a modest level of γ1 switch transcripts, data in (Toellner *et al.*, 1998) and IgG1 plasma cells (Fig. 4). Consequently, it is plausible that the increased level of γ1 switch transcript production and number of IgG1 plasma cells produced when the antigens are given together represents an adjuvant effect of the Th1 antigen—pertussis—on the Th-2 response to NP-CGG. This conclusion appears inconsistent with the hypothesis that differentiation to Th1 or Th2 depends on the cytokine environment in which T cells are primed or dendritic cells are activated to take up antigen. In addition the concept that Th1 or Th2 activity is acquired during extended proliferation of CD4 T cells in the presence of type-1 or type-2 cytokines is not supported by the rapid production of cytokine and switch transcripts.

The extra-follicular NP- and CGG-specific antibody response continues to be associated with switching to IgG1 even though the addition of pertussis is associated with the production of substantial numbers of IgG2a plasma cells.

Evidence for the development of discrete Th1 and Th2 activity in response to mixed Th1 and Th2 antigens is provided by analysis of switched NP specific plasma cells produced in the response to NP-CGG plus pertussis.

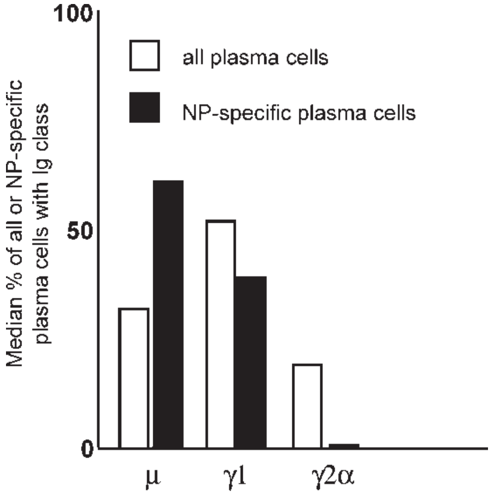

FIGURE 5 Median proportion of plasma cells that still expressed IgM or had switched to different immunoglobulin subclasses 5 days after immunization with NP-CGG in the presence of *pertussis*. 34% of all plasma cells developing in this response switched to IgG2a. However, in the plasma cells specific for NP, less than 1% switched to IgG2a and most switching occured to IgG1.

These data show that the IgG1 dominance of switching shown in the response to NP-CGG alone (Fig. 5) is retained in the responses to NP-CGG plus pertussis (Fig. 5).

### DISCUSSION

There are three main conclusions that can be drawn from the present study: (a) The onset of type-1 and type-2 cytokine production in responses to NP-CGG, or pertussis, or both of these combined coincides with the start of T cell proliferation. This contrasts with the rather slow commitment to Th1 or Th2 cytokine production seen when naïve T cells stimulated through their TCR are, respectively, co-cultured with γ-interferon or IL-4 *in vivo*.

In this report, we show that the priming of T cells after administration of an alum-precipitated haptenated-protein antigen and the onset of IL-4 and switch transcript production start around the time T cells begin to proliferate. If the adjuvant heat-killed *B. pertussis* is also given at the time of NP-CGG immunization then the time of T cell priming is accelerated. The IL-4 response in all cases is induced by the alum-precipitated NP-CGG for if *B. pertussis* is administered alone then the level of IL-4 production detected is less than 10% of that observed when both antigens are administered. Also the B cell response to NP-CGG is still Th2 like, which shows that IFN-γ was not induced in the CGG specific T cells (data not shown; Toellner *et al.*, 1998). Similarly the presence of the *B. pertussis* is likely to account for the observed upregulation of IFN-γ on day 3 when both antigens are given together. Importantly γ1 switch transcripts were upregulated to a similar level irrespective of the presence or absence of *B. pertussis*. This suggests that although the presence of this adjuvant may augment the response it is unlikely to alter the extent of the response. Thus, it is evident that in this mixed response that within the milieu there are both type-1 and type-2 cytokines at the time that T helper cell differentiation is occurring. In spite of this the specific anti-NP-CGG response remains polarized along the Th-2 pathway as is observed by the IL-4 upregulation, γ1 switch transcript production and early NP-specific IgG1 antibody production (Toellner *et al.*, 1998). Thus, it suggests that there is more involved in deciding which T helper fate a differentiating T cell will have than just the surrounding cytokine environment. Additional data were presented to the Germinal Center Conference that although the major output from a Th-2 response is the production of IL-4 this cytokine is not necessary for the induction of Th2 activity in naïve T cells as they are primed.

### *References*

Coffman, R.L., Lebman, D.A. and Rothman, P. (1993) "Mechanism and regulation of immunoglobulin isotype switching", *Adv. Immunol.* **54**, 229–270.

Collins, J.T. and Dunnick, W.A. (1993) "Germline transcripts of the murine immunoglobulin gamma 2a gene: structure and induction by IFN-gamma", *Int. Immunol.* **5**, 885–891.

Guermonprez, P., Valladeau, J., Zitvogel, L., Thery, C. and Amigorena, S. (2002) "Antigen presentation and T cell stimulation by dendritic cells", *Ann. Rev. Immunol.* **20**, 621–667.

Gulbranson-Judge, A. and MacLennan, I. (1996) "Sequential antigen-specific growth of T cells in the T zones and follicles in response to pigeon cytochrome c", *Eur. J. Immunol.* **26**, 1830–1837.

Janeway, C.A. Jr, and Medzhitov, R. (1998) "Introduction: the role of innate immunity in the adaptive immune response", *Semin. Immunol.* **10**, 349–350.

Khair, O.A., Davies, R.J. and Devalia, J.L. (1996) "Bacterial-induced release of inflammatory mediators by bronchial epithelial cells", *Eur. Respir. J.* **9**, 1913–1922.

Luther, S.A., Gulbranson-Judge, A., Acha-Orbea, H. and MacLennan, I.C. (1997) "Viral superantigen drives extrafollicular and follicular B cell differentiation leading to virus-specific antibody production", *J. Exp. Med.* **185**, 551–562.

Medzhitov, R. (2001) "Toll-like receptors and innate immunity", *Nature Rev. Immunol.* **1**, 135–145.

Nossal, G.J. and Karvelas, M. (1990) "Soluble antigen abrogates the appearance of anti-protein IgG1-forming cell precursors during primary immunization", *Proc. Natl Acad. Sci. USA* **87**, 1615–1619.

Svetic, A., Finkelman, F.D., Jian, Y.C., Dieffenbach, C.W., Scott, D.E., McCarthy, K.F., Steinberg, A.D. and Gause, W.C. (1991) "Cytokine gene expression after *in vivo* primary immunization with goat antibody to mouse IgD antibody", *J. Immunol.* **147**, 2391–2397.

Toellner, K.M., Gulbranson-Judge, A., Taylor, D.R., Sze, D.M. and MacLennan, I.C. (1996) "Immunoglobulin switch transcript production *in vivo* related to the site and time of antigen-specific B cell activation", *J. Exp. Med.* **183**, 2303–2312.

Toellner, K.M., Luther, S.A., Sze, D.M., Choy, R.K., Taylor, D.R., MacLennan, I.C.M. and Acha-Orbea, H. (1998) "T helper 1 (Th1) and Th2 characteristics start to develop during T cell priming and are associated with an immediate ability to induce immunoglobulin class switching", *J. Exp. Med.* **187**, 1193–1204.

Weible, E.R. (1963) "Principle and methods for the morphometric study of the lung and other organs", *Lab. Investig.* **12**, 131–135.

